# A Unique Presentation of Anti-RNA Polymerase III Positive Systemic Sclerosis Sine Scleroderma

**DOI:** 10.1155/2016/8536341

**Published:** 2016-07-31

**Authors:** Cody M. Lee, Diana Girnita, Arundhati Sharma, Surabhi Khanna, Jean M. Elwing

**Affiliations:** ^1^Department of Internal Medicine, University of Cincinnati, Cincinnati, OH 45267, USA; ^2^Division of Immunology, Allergy and Rheumatology, University of Cincinnati, Cincinnati, OH 45267, USA; ^3^Department of Internal Medicine, The Christ Hospital, Cincinnati, OH 45219, USA; ^4^Division of Pulmonary, Critical Care, and Sleep Medicine, Department of Internal Medicine, University of Cincinnati, Cincinnati, OH 45267, USA

## Abstract

Systemic sclerosis is a rare autoimmune disorder with a wide spectrum of clinical manifestations and a multitude of autoantibodies that are associated with it. In the past several years, advances in serologic testing have led to research indicating important prognostic and phenotypic associations with certain subsets of autoantibodies. In particular, anti-RNA polymerase III (anti-RNAP III) has been associated with diffuse cutaneous disease, scleroderma renal crisis, a temporal relationship with malignancy, myositis, synovitis, joint contractures, and gastric antral vascular ectasia. However, anti-RNAP III has not been associated with systemic sclerosis sine scleroderma. We describe a patient with an atypical presentation of anti-RNAP III positive systemic sclerosis sine scleroderma who presented without the typical features of anti-RNAP III disease. Instead, she presented with critical digital ischemia, pulmonary arterial hypertension, gastroesophageal reflux disease, interstitial lung disease, and no clinically detectable sclerodactyly.

## 1. Introduction

Systemic sclerosis (SSc) is an uncommon generalized connective tissue disorder with a broad spectrum of clinical diseases. Subtypes of the disease include diffuse cutaneous systemic sclerosis (dcSSc), limited cutaneous systemic sclerosis (lcSSc), systemic sclerosis sine scleroderma (ssSSc), and overlap disease. There are numerous autoantibodies associated with the disease, each of which can have important prognostic and phenotypic associations. One such autoantibody is anti-RNA polymerase III (anti-RNAP III). This specific autoantibody has been associated with diffuse cutaneous disease, scleroderma renal crisis, a temporal relationship with malignancy, myositis, synovitis, joint contractures, and gastric antral vascular ectasia [1, 2]. However, anti-RNAP III has not been described as being associated with ssSSc nor is it typically associated with pulmonary arterial hypertension (PAH) [2, 3].

## 2. Case Description

**Figure 1:**
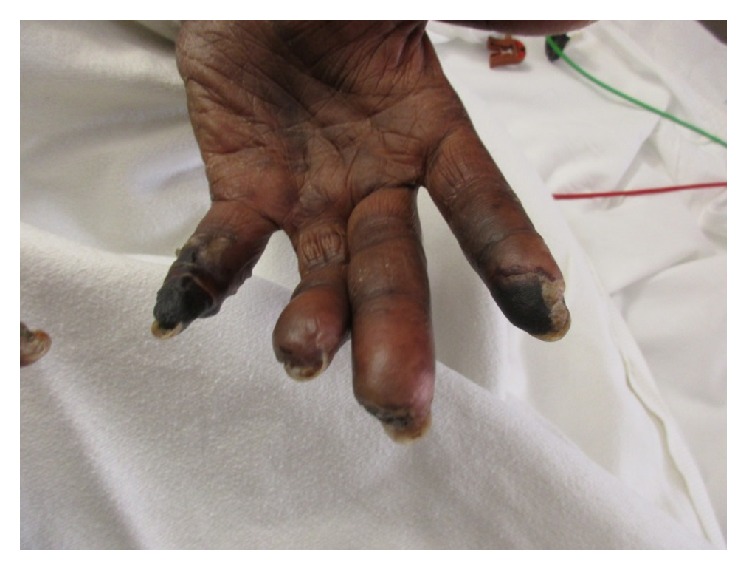
Left hand on admission.

**Figure 2:**
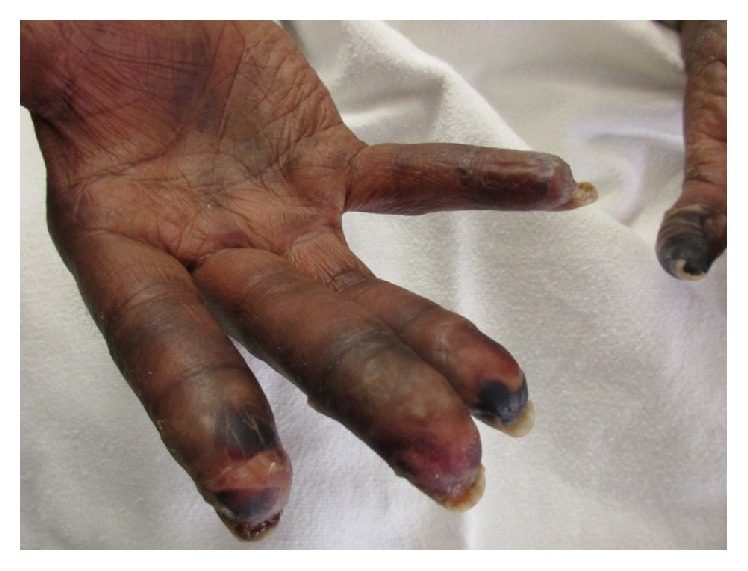
Right hand on admission.

A 59-year-old African American female with a history of hypertension, gastroesophageal reflux disease, and Raynaud's phenomenon (RP) initially presented to an outside facility with 6 months of progressive shortness of breath, dry cough, and worsening RP with digital ulceration. At that time, her workup was notable for a CT scan of her chest showing fibrotic interstitial lung disease (ILD) most consistent with a non-specific interstitial pneumonia pattern and right heart catheterization showing pulmonary artery pressures of 80/32 mmHg (mean 47 mmHg), pulmonary capillary wedge pressure of 9 mmHg, cardiac output of 4.91 L/minute, and pulmonary vascular resistance of 619 dyne·second·cm^−5^. Based on these findings, she was started on treatment with sildenafil 40 mg three times daily and mycophenolate mofetil 500 mg daily and referred to our PAH clinic for further evaluation. In clinic, she was noted to have progression of her pulmonary symptoms and dry gangrene (Figures 1 and 2). Given her clinical decline, she was admitted for initiation of epoprostenol as well as further workup for the underlying etiology of her symptoms.

On admission, the physical exam was notable for multiple gangrenous digits, a right fifth digital ulcer, accentuated P2, 2/6 systolic murmur at the right upper sternal border, and coarse crackles bilaterally on lung auscultation. Given the extent of the gangrene, examination of the hands was limited. Her digits did appear slightly puffy; however, there did not appear to be any sclerodactyly or any other skin involvement. Nailfold capillaroscopy done using the ophthalmoscope was notable for capillary dilatations and dropout in the nongangrenous digits. An extensive laboratory workup was completed with pertinent positives including an ANA (1 : 320 nucleolar pattern) and anti-RNAP III (Tables 1 [Table tab2] [Table tab3]–4). Notably, there was no evidence of other SSc autoantibodies, antiphospholipid antibodies, or renal involvement. Her initial CK on admission was elevated to 1162; however, it quickly downtrended over the next several days without any targeted treatment and the patient had no weakness noted. It was thought that the elevation in CK was likely secondary from the degree of critical digital ischemia. Further workup included pulmonary function tests showing a restrictive lung defect with an FVC of 2.15 L (64% predicted), FEV1/FVC ratio of 69%, DLCO of 7.98 mL/mmHg·sec (37% predicted), and an FVC/DLCO ratio of 1.7. In addition, an esophagram was done showing mild dilation and dysmotility of the distal esophagus and a cardiac MRI showing no evidence of infiltrative disease. Lastly, a VQ scan revealed a large unmatched perfusion defect of the left base consistent with pulmonary embolism (PE), but this was not felt to be the sole etiology for her PAH. The etiology of her PE was not elucidated at that time. However, her hypercoagulable workup was negative (Table 2) and CT images of her chest, abdomen, pelvis, and head showed no evidence of any malignancy, although it should be noted that she was not up to date on her age appropriate cancer screening including a colonoscopy, pap smear, and mammogram. Further workup was to be pursued as an outpatient given a low suspicion at the time for an underlying malignancy. Given the above findings, she fulfilled the 2013 ACR criteria for a diagnosis of SSc with a score of 12 and she was diagnosed with ssSSc.

For her treatment, she was started on a continuous heparin and epoprostenol infusion which was titrated up to 7 ng·kg^−1^·min^−1^ with further increases as an outpatient. She was continued on sildenafil 40 mg three times daily as well as mycophenolate mofetil, which was increased to 1 g twice daily by discharge. Her pulmonary symptoms significantly improved as did her digital ulceration and pain.

## 3. Discussion

The patient presented for discussion represents a unique case of anti-RNAP III positive ssSSc. Systemic sclerosis sine scleroderma is a rare subset of scleroderma accounting for approximately 2–9% of SSc patients [1, 4]. These patients are noted to have internal organ involvement, laboratory features, and serum autoantibody type similar to those with lcSSc but lack the characteristic skin thickening noted in other forms of SSc. Prior studies have characterized these patients as having predominately pulmonary, gastrointestinal, and peripheral vascular involvement. Notably they often lack renal involvement and may have higher rates of PAH as compared to their lcSSc counterparts [1, 5].

Based on a recent multicenter study, serological profiles of patients with ssSSc were similar to those with lcSSc but different from dcSSc with higher rates of anti-centromere antibodies (50% ssSSc versus 12% in dcSSc) and lower rates of anti-topoisomerase I antibodies (16% ssSSc versus 22% dcSSc). Yet, no patients in this cohort presented with anti-RNAP III (0% ssSSc versus 35% dcSSc) [3]. Despite some similarities with our patient's presentation to typical ssSSc disease, the major outlier is the anti-RNAP III positivity. Typically, anti-RNAP III is associated with increased risk of developing scleroderma renal crisis, diffuse skin disease, a temporal relationship with malignancy, gastric antral vascular ectasia, myositis, synovitis, and joint contractures, none of which were seen in our patient [2, 6]. Other features such as PAH are similarly not common in the anti-RNAP III positive groups as shown in a recent study of 164 patients with PAH related SSc. This study noted that only 9% were anti-RNAP III positive with the larger portion being 37% anti-centromere antibody (ACA) and 24% ANA (anti-nucleolar pattern) [7]. Furthermore, based on a 20-year prospective study, patients with RP, anti-RNAP III positivity, and abnormal nailfold capillaroscopy are 60 times more likely to develop dcSSc versus other forms of SSc [8].

Beyond the phenotypic variation between autoantibody profiles, there is also significant variation of autoantibodies based on ethnicity. This has been highlighted in several prior studies showing that Caucasians typically are positive for anti-RNAP III, ACA, and anti-Pm/Scl, while African Americans have more anti-U3 RNP, anti-U1 RNP, and anti-topoisomerase I [9–11]. In addition, a prior study of 1432 SSc patients and their autoantibody status showed that only 4% of the anti-RNAP III positive group was African American with the overwhelming majority being Caucasian [9].

In conclusion, to our knowledge, this is the first case in the literature of an ssSSc patient that is anti-RNAP III positive who presented with digital ischemia, PAH, and ILD without the typical features of anti-RNAP III disease. We identified only one other case report of an ssSSc patient that was anti-RNAP III positive. This patient, however, presented with 6 months of symmetric polyarthritis and rapidly progressive renal failure [12].

## Figures and Tables

**Table 1:** Autoimmune laboratory results.

Labs	Value	Normal range
ESR	120 mm/hr	0–29 mm/hr
CRP	8.8	1.0–10.0 mg/L
Creatine kinase	1162	30–223 U/L
ANA ratio	2.85	0.00–0.99
Nucleolar pattern	1 : 320	1 : 40
ANCA myeloperoxidase	<0.9	0.0–9.0 U/mL
ANCA proteinase 3	<3.5	0.0–3.5 U/mL
Atypical P-ANCA	<1 : 20	Negative < 1 : 20 titer
Anti-centromere antibody	<0.2	0.0–0.9 AI
Double stranded DNA	1	0–9 IU/mL
C3 complement	139	87–200 mg/dL
C4 complement	29	19–52 mg/dL
Anti-Jo-1	Negative	Negative
SCL-70 antibody	Negative	Negative
SM antibody	Negative	Negative
SM/RNP antibodies	Negative	Negative
Anti-SSA/SSB antibodies	Negative	Negative
Anti-RNA polymerase III antibody	33.6	<20 units/mL
Anti-Th/To	Negative	Negative
Anti-U3RNP	Negative	Negative
Anti-U1RNP	<20	<20

ESR: erythrocyte sedimentation rate; CRP: C-reactive protein; ANA: anti-nuclear antibody; ANCA: anti-neutrophil cytoplasmic antibodies.

**Table 2 tab2:** Hypercoagulable laboratory results.

Labs	Value	Normal range
Anticardiolipin antibody	Negative	Negative
Factor V Leiden	Normal	Normal
Homocysteine	12.1	5.0–12.0 *μ*mol/L
Lupus anticoagulant	Negative	Negative
Beta-2 glycoprotein 1 IgG	<9	0–20 GPI IgG units
Beta-2 glycoprotein 1 IgM	<9	0–32 GPI IgM units
Beta-2 glycoprotein 1 IgA	9	0–25 GPI IgA units
Protein C activity	156	70–130%
Protein S activity	55	55–123%
Gamma globulin	2.8	0.50–1.50 g/dL
Kappa/lambda ratio	1.70	0.26–1.65 ratio
M spike	0.0	None

**Table 3 tab3:** Other laboratory results.

Labs	Value	Normal range
White blood cell count	8.2	3.8–10.8 10*E*3/*µ*L
Hemoglobin	11.2	11.7–15.5 g/dL
Hematocrit	35.6	35–45%
MCV	91.4	80–100 fL
Platelets	410	140–400 10*E*3/*µ*L
Sodium	135	133–146 mmol/L
Potassium	3.8	3.5–5.3 mmol/L
Chloride	101	98–110 mmol/L
Creatinine	0.44	0.60–1.30 mg/dL
BUN	13	7–25 mg/dL
Glucose	93	70–100 mg/dL
Calcium	10	8.6–10.3 mg/dL
Total bilirubin	0.3	0–1.5 mg/dL
Direct bilirubin	0.06	0–0.40 mg/dL
AST	24	13–39 U/L
ALT	16	7–52 U/L
Alkaline phosphatase	50	36–125 U/L
Total protein	7.1	6.4–8.9 g/dL
Albumin	3.5	3.5–5.7 g/dL

**Table 4:** Urinalysis.

Labs	Value	Normal range
Specific gravity	1.013	1.005–1.035
pH	6	5–8
Protein	Negative	Negative
Glucose	Negative	Negative
Blood	Negative	Negative
Nitrite	Negative	Negative
Leukocytes	Negative	Negative
